# Third ventricular choroid plexus papilloma: a pure neuroendoscopic approach

**DOI:** 10.3171/2023.1.FOCVID22145

**Published:** 2023-04-01

**Authors:** Preston D’Souza, Thomas S. Frank, Aaron Mohanty

**Affiliations:** Department of Neurosurgery, The University of Texas Medical Branch, Galveston, Texas

**Keywords:** neuroendoscope, NICO Myriad, choroid plexus papilloma

## Abstract

Pure endoscopic technique in resection of intraventricular tumors is an emerging technology. This case demonstrates resection of a multicentric choroid plexus papilloma in a 2-month-old child. This child had two district tumors: one located in the left atrium and another in the third ventricle. Initial microsurgery was performed to resect the left atrial tumor. With the tumor noted to be not very vascular at initial surgery, the third ventricle tumor was resected with a GAAB neuroendoscope and NICO Myriad. A gross-total resection was achieved. At 3 years’ follow-up, the child remains tumor free and developing without any functional deficits.

The video can be found here: https://stream.cadmore.media/r10.3171/2023.1.FOCVID22145

## Transcript

Hello. Today we will discuss a third ventricular choroid plexus papilloma resection through a pure neuroendoscopic approach. The case begins with a 2-month-old male who was found to have a head size in the 99th percentile during a well-child check. The patient developed some lateral gaze deviation but was otherwise neurologically intact with no developmental abnormalities nor issues meeting milestones thereafter. Preoperative imaging revealed multicentric contrast-enhancing lesions involving the third ventricle, left lateral ventricle, and left atrium as well as ventriculomegaly.

### 1:00 First Stage of Resection.

The first stage of resection involved a left temporoparietal craniotomy to remove the left atrial component.^1^ An open approach was utilized due to the unknown vascularity of the tumor. A postoperative external ventricular drain was placed, and pathology was consistent with an atypical choroid plexus papilloma. Immediate postoperative scan revealed resection of the left atrial component, retention of the third ventricular component, and relatively unchanged sizes of ventricles.

### 1:38 Second Stage of Resection.

Three days later, a neuroendoscopic approach was utilized through a GAAB pediatric neuroendoscope and NICO Myriad system for debulking.^2^ A left frontal burr hole was used for entry point, and hemostatic control was utilized by pressure irrigation and bipolar cautery coupled together. Additionally, the trajectory of the endoscope was rather facile after its entry through a left frontal burr hole which was a centimeter anterior to the coronal suture. The endoscope was placed perpendicular to the brain, and after its entry through the foramen of Monro, the endoscope could be angled steeply both anteriorly and posteriorly without issue to access the entirety of the third ventricle.

### 2:34 Entry Into Left Lateral Ventricle.

So here is entry into the left lateral ventricle. Here is our septum with septostomy from the external ventricular drain and the choroid plexus papilloma in front of us with the characteristic cauliflower shape.

### 2:43 Resection of Tumor.

Resection ensues with the NICO Myriad system. The soft-tissue aspirating system was very useful to debulk this relatively avascular tumor.^2^ The goal of this surgery was to follow the tumor along the left lateral ventricle, through the foramen of Monro, and into the third ventricle.

### 3:08 Control Bleeding.

While remaining relatively avascular, few islands of vascularity were encountered such as here. If a bleeding vessel is encountered, copious pressure irrigation with lactated Ringer’s is performed. Additionally, bipolar cautery is utilized to fully ameliorate the bleeding. Once confirmation is obtained that the bleeding is controlled and our field of view is unobscured, the NICO Myriad can be brought back into field of view and resection can ensue.

### 3:41 Entry Into Third Ventricle.

So here is entry into the third ventricle. Below is the floor of the third ventricle, the tuber cinereum, pulsations of the basilar artery, and the mamillary bodies identified.

### 3:58 Finding the Aqueduct.

Working more posteriorly, the aqueduct can be identified after more resection, and below on the screen is the superior component of the third ventricle showing the internal cerebral veins.

### 4:22 Confirmation of Resection Extent.

Once resection is complete, confirmation of a gross-total resection can be achieved with a neuroendoscope. Again, we can see the pulsations of the basilar artery, the mamillary bodies, and an unobstructed cerebral aqueduct.

### 4:41 Postoperative Outcome.

Postoperative MRI revealed a gross-total resection with some residual blood product. The patient remained neurointact, gaze deviation improved, there were no postoperative events worrisome for acute hydrocephalus, and the subdural collections were not unexpected given extent of initial ventriculomegaly. Three years later, MRI revealed stability of the gross-total resection, resolution of the postoperative subdural collections, and stable ventricle sizes.

### 5:16 Conclusions.

The neuroendoscopic method in conjunction with the NICO Myriad was particularly useful to both access the third ventricle as well as debulk this tumor.^3–6^ While the NICO Myriad was our instrument of choice, it should be noted that there are other options for tumor debulking, such as the endoscopic ultrasonic aspirator.^7^ Hemostatic control was achieved with pressure irrigation and bipolar cautery throughout the case. The neuroendoscopic method allowed the advantage of direct visualization for direct tumor resection and confirmation of an unobstructed aqueduct. All of this was able to be achieved with minimal contusion to surrounding brain structures. The patient still remains to do well to this day with no overt neurological deficits. It should also be noted that an alternative approach could have been taken for this tumor. A single-staged craniotomy in which the left lateral ventricle is accessed through the superior parietal lobule followed by a subchoroidal resection through the third ventricle could have been done in one stage. The decision to pursue a two-stage surgery was mainly influenced by the unknown vascularity of the tumor as well as the balance and rapidity of blood loss for a child in a single-stage craniotomy.

### 6:36 Disclosure and References.

Thank you for listening to our video. As a final disclosure, we have no conflicts of interests nor any financial endorsements to any of the devices listed in this video.

## Disclosures

The authors report no conflict of interest concerning the materials or methods used in this study or the findings specified in this publication.

## Author Contributions

Primary surgeon: Mohanty. Assistant surgeon: Frank. Editing and drafting the video and abstract: D’Souza. Critically revising the work: Mohanty, D’Souza. Reviewed submitted version of the work: D’Souza. Approved the final version of the work on behalf of all authors: Mohanty. Supervision: Mohanty.

## Supplemental Information

### Patient Informed Consent

The necessary patient informed consent was obtained in this study.
